# Histopathological Study of Skin Lesions in a Tertiary Care Hospital: A Descriptive Cross-sectional Study

**DOI:** 10.31729/jnma.4799

**Published:** 2020-04-30

**Authors:** Sanat Chalise, Ramesh Dhakhwa, Sailesh Bahadur Pradhan

**Affiliations:** 1 Department of Pathology, Kathmandu Medical College and Teaching Hospital, Sinamangal, Kathmandu, Nepal

**Keywords:** *dermatitis*, *papular*, *tumors*

## Abstract

**Introduction:**

Skin diseases are much common in developing countries. The spectrum varies according to geographic distribution, gender, age, and coexisting disorder. We conducted this study to find out the prevalence of different skin lesions and to evaluate their frequency and site of distribution.

**Methods:**

A descriptive cross-sectional study was done in the pathology department of Kathmandu Medical college from June 2019 to November 2019 after ethical clearance. The skin biopsies were processed, sectioned and stained with Haematoxylin and eosin and evaluated. A convenience sampling method was used. Data was collected and entry was done in Statistical Packages for Social Services version 20.0, point estimate at 95% Confidence Interval was calculated along with frequency and proportion for binary data.

**Results:**

Among 133 skin biopsies examined, noninfectious vesicobullous and vesicopustular disease were found in 42 (46.6%) cases followed by microbial disease in 22 (24.5%) and noninfectious erythematous papular and squamous disease in 21 (23.4%) cases. Spongiotic dermatitis was the most common vesicobullous disease seen in 26 (28.9%) cases. Leprosy was the commonest microbial disease found in 7 (7.8%) cases. The commonest noninfectious erythematous papular and squamous disease was erythema dyschromicum perstans seen in 7 (7.8%) cases. The commonest neoplastic lesion was keratinocytic tumor seen in 12 (32.5%) cases. The commonest tumor of the skin was intradermal nevus seen in 6 (16.3%) cases. Upper extremities were the most frequently involved site by skin lesions.

**Conclusions:**

Spongiotic dermatitis is a predominating non-neoplastic and overall skin lesion which was similar to the other studies done. Histopathological examination is the gold standard for the proper diagnosis as histomorphological features distinguish various skin lesions.

## INTRODUCTION

Skin diseases affect all age groups and are much common in developing countries. In the field of dermatology, 2000 different skin diseases are well known.^1^ The pattern of skin disease varies from country to country and region to region within the same country. Various factors such as racial, environment and social customs influence skin disease.^2^

Skin biopsies are often performed as many of the diseases have clinical overlaps which range from simple acne to serious disorder like toxic epidermal necrolysis and neoplastic condition.^1,3^ The clinically different skin lesions may show similar histologic findings, therefore, a correlation between clinical presentation and history with histopathological findings improves the diagnostic specificity of the skin lesions.^4^

This study was done to find out the prevalence of various skin lesions and their frequency as well as site of distribution.

## METHODS

This descriptive cross-sectional study was conducted among the patients visiting Kathmandu Medical College Public Limited, Sinamangal, Nepal from June 2019 to November 2019. The ethical approval for the study was taken from the Institutional Review Committee of Kathmandu Medical College Teaching Hospital, with reference number 3105201114. Data was collected from the patients from whom biopsies of skin lesions had been taken. All the patients who were subjected to skin biopsy were included in this study. Inadequate skin biopsies and cystic skin lesions were excluded from the study. The biopsies taken were fixed in 10% formalin and then processed. Four microns thick sections were taken and stained with Haematoxylin and Eosin stain (H&E). Special stains like Ziehl- Neelsen (ZN), Periodic Acid Schiff (PAS) and Fite-Faraco were used whenever required. Convenient sampling was done and sample size was calculated using the following formula.

$$\begin{array}{ll} \text{n} & \text{= }{\text{Z}}^{\text{2}}\times \text{p }\times \text{q}/{\text{e}}^{\text{2}}\\ & \text{= }{(\text{1.96)}}^{\text{2}}\times \text{(}0.\text{096)}\times (1-0.096)/{\text{(0.05)}}^{\text{2}}\\ & \text{= }133 \end{array}$$

where,

n = sample size
p = prevalence of 9.6 %
q = 1-p
e = margin of error (5%)
Z = 1.96 at 95% CI

The data was entered in SPSS (Statistical Packages for Social Services) version 20.0. The descriptive statistical analysis was done.

## RESULTS

Out of 133 skin biopsies, 90 (67.7%) were non-neoplastic and 37 (27.8%) were neoplastic. Histopathological diagnosis was inconclusive in 6 (4.5%) cases (Table 1).

**Table 1:** Types of skin lesions based on histopathology.

S.N.	Skin lesion	n (%)
1.	Non-neoplastic	90 (67.7)
2.	Neoplastic	37 (27.8)
3.	Inconclusive	6 (4.5)
	Total	133 (100)

The most common non-neoplastic histopathological pattern observed was noninfectious vesicobullous and vesicopustular disease comprising of 42 (46.6%) cases followed by microbial disease 22 (24.5%) cases and noninfectious erythematous papular and squamous disease 21 (23.4%) cases. Connective tissue disease was the least commonly seen in 5 (5.5%) cases. The most common vesicopustular disease was spongiotic dermatitis comprising 26 (28.9%) cases followed by lichen simplex chronicus seen in 10 (11.1%) cases. Leprosy was the commonest microbial disease seen in 7 (7.8%) cases followed by verruca in 6 (6.7%) and dermatophytosis seen in 4 (4.5%) cases. Among noninfectious erythematous papular and squamous disease, erythema dyschromicum perstans was the commonest disease seen in 7 (7.8%) cases followed by lichen planus seen in 5 (5.6%) cases (Table 2).

**Table 2:** Distribution of cases according to histopathological patterns of non-neoplastic.

S.N.	Skin lesions	n (%)
	Non-infectious vesicobullous and vesicopustular disease	42 (46.6)
1.	Spongiotic dermatitis	26 (28.9)
2.	Lichen simplex chronicus	10 (11.1)
3.	Pemphigus	5 (5.5)
4.	Subepidermal bullous disease	1 (1.1)
	Non-infectious erythematous papular and squamous disease	21 (23.4)
5.	Erythema Dyschromicum perstans	7 (7.8)
6.	Lichen planus	5 (5.6)
7.	Psoriasis	4 (4.5)
8.	Pityriasis rosea	2 (2.2)
9.	Lichen planus pigmentosus	2 (2.2)
10.	Urticaria	1 (1.1)
	Microbial disease	22 (24.5)
11.	Leprosy	7 (7.8)
12.	Tuberculosis	2 (2.2)
13.	Dermatophytosis	4 (4.5)
14.	Sporotrichosis	2 (2.2)
15.	Chromoblastomycosis	1 (1.1)
16.	Verruca	6 (6.7)
	Connective tissue disease	5 (5.5)
17.	Lichen sclerosus et atrophicus	3 (3.3)
18.	Discoid lupus erythematosus	2 (2.2)
	**Total**	**90 (100)**

Among neoplastic skin lesions, keratinocytic tumor was most commonly seen in 12 (32.5%) cases followed by melanocytic tumors seen in 9 (24.3%) cases. The prevalence of appendageal tumors and soft tissue tumor was equal. Both seborrheic keratosis and squamous cell carcinoma was a commonest keratinocytic tumor seen in 4 (10.8%) cases respectively. Intradermal nevus was the commonest melanocytic and overall skin tumor observed in 6 (16.3%) cases (Table 3).

**Table 3:** Distribution of neoplastic skin lesions on the basis of histopathology.

Types of skin tumor	n (%)
Keratinocytic tumor	12 (32.5)
Seborrheic keratosis	4 (10.8)
Squamous cell carcinoma	4 (10.8)
Basal cell carcinoma	3 (8.1)
Keratoacanthoma	1 (2.7)
Melanocytic tumor	9 (24.3)
Intradermal nevus	6(16.3)
Compound nevus	2(5.4)
Malignant melanoma	1(2.7)
Appendageal tumor	8 (21.6)
Pilomatricoma	2 (5.4)
Tricholemmoma	1 (2.7)
Eccrine spiroadenoma	1 (2.7)
Nevus sebaceous	2 (5.4)
Apocrine hidrocystoma	1 (2.7)
Cutaneous lymphoma	1 (2.7)
Soft tissue tumor	8 (21.6)
Soft fibroma	3 (8.1)
Dermatofibroma	2 (5.4)
Keloid	1 (2.7)
Pearly penile papule	1 (2.7)
Dermatofibrosarcoma protuberens	1 (2.7)
Total	37 (100)

Upper extremities were the commonest site of involvement seen in 35 (26.3%) cases followed by lower extremities 30 (22.5%), trunk and abdomen 27(20.3%) and neck 17 (12.8%) cases (Table 4).

**Table 4:** Site of involvement by different skin lesions.

Site	n (%)
Scalp	6 (4.5)
Ear	2 (1.5)
Face	14 (10.5)
Neck	17 (12.8)
Trunk and abdomen	27 (20.3)
Upper extremities	35 (26.3)
Lower extremities	30 (22.5)
Penis	1 (0.8)
Vulva	1 (0.8)
Total	133 (100)

## DISCUSSION

Skin lesions are due to imbalance in homeostasis that results in conditions as diverse as wrinkles and hair loss, rashes and blisters and life-threatening cancers.^5^ A skin biopsy may not be required in all the skin lesions but for the proper diagnosis and identification of etiological agents, dermatologist used to do it.^6^

This study showed the highest frequency of skin disease in the age range of 41-50 years. In contrast to the finding of this study, Bezbaruah R et al.^5^ and Abubaker SD et al.^7^ found the highest frequency in 21-30 years whereas Adhikari RC et al.^1^ found the highest frequency in 31-40 years. The current study shows slight female preponderance which was similar to the study done by Bezbaruah R et al.^5^ and Adhikari et al.^1^ however Dayal et al.^8^ and Kumar V et al.^9^ found male predominance in their studies.

Our study showed 67.7% of non-neoplastic skin lesions which was much higher in comparison to those of neoplastic skin lesions (27.8%). However, Bezbaruah R et al,^5^ Abubaker SD et al.^7^ and Sushma et al.^6^ in their study found neoplastic lesions as a major entity. Spongiotic dermatitis (28.9%) was the most common vesicobullous disease found in our study. A similar result was also found in the study done by Adhikari et al.^1^ and Ogun GO et al.^10^ Agrawal S et al and Reddy et al found psoriasis and lichen planus as a commonest papulosquamous disease.^11,12^ However, in contrast to their studies, erythema dyschromicum perstans was a commonest papulosquamous disease found in our study.

Leprosy (7.8%) was a commonest infective skin lesion in our study followed by verruca (6.7%) and dermatophytosis (4.5%). Agrawal et al. also found leprosy as a commonest infectious skin disease in their study.^11^ In contrast to our study, previous studies done in Nepal by Karn et al.^13^ and Walker et al.^14^ found dermatophytosis as the commonest infective skin lesion and they concluded that hot and humid climatic conditions in a certain geographic region may be the possible cause for the increase in prevalence in fungal infections.

The common neoplastic lesion observed in our study was keratinocytic tumor (32.5%) followed by the melanocytic tumor (24.3%). However, the overall commonest lesion was intradermal nevus. Among keratinocytic tumors, both seborrheic keratosis (10.8%) and squamous cell carcinoma (10.8%) share an equal number of cases. These findings were comparable to the study done by Thapa et al.^15^ and Rauniyar et al.^16^

In our study, an inconclusive result was obtained in about 4.5% of cases. This result was similar to the study done by Adhikari et al.^1^ and Barman DD et al.^17^

The skin lesions were commonly seen in the upper and lower extremities in our study. Adhikari et al. in their study also found upper and lower extremities as the commonest site of involvement by skin lesions.^1^ However, in contrast to our study, Bezbaruah R et al. found eyelid and lip as a frequent site of involvement.^5^

## CONCLUSIONS

Prevalence of spongiotic dermatitis was higher which was similar to the other studies done. We observed a wide spectrum of skin lesions ranging from dermatitis to malignant neoplasm. The importance of specific histomorphological features lies in distinguishing various skin lesions and play a major role in making the final diagnosis of these diverse skin lesions. This highlights the role of histopathological examination for the proper management of patient.

## References

[1] Adhikari RC, Shah M, Jha AK (2019). Histopathological spectrum of skin diseases in a tertiary skin health and referral centre. J Pathol Nep.

[2] Singh A, Nirmal AK, Gupta P, Jha JK (2018). Histopathological review of dermatological disorders with a keynote to granulomatous lesions: A Retrospective Study. Ind J Pub Health Res Dev.

[3] Umarji S, Ravikumar G, Antony M, Tirumalae R (2018). Comparison of clinical diagnosis with histopathology in inflammatory skin diseases: a retrospective study of 455 cases. Egypt J Dermatol Venerol.

[4] Tayal DA, Bharti DR, Kumar DH (2019). Study of histomorphological pattern in non-infectious erythematous papulosquamous lesion of skin Atterriary Care Hospital, Up. IOSR-JDMS.

[5] Bezbaruah R, Baruah M (2018). Histopathological spectrum of skin lesions-A hospital based study. Indian J Appl Res.

[6] Sushma C (2018). Histomorphological motif of skin lesions - A model analysis in a tertiary care teaching hospital. IOSR-JDMS.

[7] Abubakar SD, Tangaza AM, Sahabi SM, Legbo JN (2016). Histopathological pattern of skin lesion in Usmanu Danfodiyo University Teaching Hospital, Sokoto, Nigeria. Afr J Cell Path.

[8] Dayal SG, Gupta GD (1977). A Cross Section of Skin Diseases in Bundelkhand Region, UP. Indian J Dermatol Venereol Leprol.

[9] Kumar V, Goswami HM (2018). Spectrum of Non-neoplastic Skin Lesions: A Histopathological study based on punch biopsy. Int J Cur Res Rev.

[10] Ogun GO, Okoro OE (2016). The spectrum of non-neoplastic skin lesions in Ibadan, Nigeria: a histopathologic study. Pan Afr Med J.

[11] Agrawal S, Mishra KB, Gupta CM (2018). Histopathological spectrum of non-infectious erythematous, papulo-squamous lesions: at a teritary care institute. Int J Res Med Sci.

[12] Reddy BR, M NKrishna (2014). Histopathological spectrum of non-infectious erythematous, papulo-squamous lesions. Asian Pac J Health Sci.

[13] Karn D, Khatri R, Timalsina M (2010). Prevalence of Skin Diseases in Kavre District, Nepal. Nepal J Dermatol Venereol & Leprol.

[14] Walker SL, Shah M, Hubbard VG, Pradhan HM, Ghimire M (2007). Skin disease is common in rural Nepal: results of a point prevalence study. Br J Dermatol.

[15] Thapa R, Gurung P, Hirachand S, Shrestha SB (2018). Histomorphologic profile of skin tumors. J Nepal Med Assoc.

[16] Rauniyar SK, Agarwal A (2003). Histomorphologic pattern of skin lesions in Kathmandu valley: a retrospective study. Nepal Med Coll J.

[17] Barman DD, Bhattacharyya P, Ray PS, Sarkar S, Sarkar R, Roy AK (2018). Clinicopathological correlation of noninfectious erythematous papulosquamous cutaneous lesions in a tertiary care hospital. Indian J Dermatopathol Diagn Dermatol.

